# Editorial: Impact of climate change on coastal environmental variability and aquatic physiology

**DOI:** 10.3389/fphys.2023.1305645

**Published:** 2023-10-27

**Authors:** Folco Giomi, Gisela Lannig, Marco Fusi

**Affiliations:** ^1^ Independent Researcher, Padova, Italy; ^2^ Integrative Ecophysiology, Alfred Wegener Institute Helmholtz Center for Polar & Marine Research, Bremerhaven, Germany; ^3^ Joint Nature Conservation Committee, Peterborough, United Kingdom

**Keywords:** coastal ecosystems, climate change, aquatic physiology, environmental variability, marine life resilience, climate-induced fluctuations

## Introduction

The ocean is experiencing a profound transformation driven by anthropogenic-induced climate change (Rosa et al., 2014; Frazão Santos et al., 2020). Within this global narrative, coastal ecosystems emerge as crucibles of change, bearing the imprint of both human activity and climatic shifts. Coastal habitats, characterized by their intricate interplay of environmental parameters and energetic flows, are particularly vulnerable to the convergence of these forces (Giomi et al., 2023). As we stand on the precipice of unprecedented change, the Research Topic *Impact of climate change on coastal environmental variability and aquatic physiology* in Frontiers in Physiology beckons us to delve into the intricate dynamics of these ecosystems and their symbiotic relationship with marine life.

In coastal environments, the rhythmic oscillation of environmental variables as for example the water flow controlled by the tidal regime, the diel oxygen fluctuations by photosynthetic activity can be disrupted by to stochastic event like marine heatwaves conditioning the lives of countless organisms. The ocean currents, tides, and temperature gradients that characterize these habitats provide the backdrop against which marine life has evolved and adapted (Fusi et al., 2023). However, the relentless advance of climate change is now amplifying the complexity of these rhythms. Coastal ecosystems, shaped by eons of natural variability, are grappling with an unprecedented pace of change as human activity alter the climate (Bitter et al., 2021).

Amidst these fluctuations, aquatic physiology emerges as a linchpin linking the fate of organisms to the evolving coastal environment. The physiological responses of marine life are both the canaries in the coalmine, signaling environmental stress, and the resilient echoes of life’s adaptability (Giomi et al., 2019; Booth et al., 2021). This intricate and constant interplay between physiology and environment shapes the very fabric of coastal ecosystems. From the microscopic to the macroscopic, organisms have honed an array of physiological mechanisms to sense, respond, and even anticipate the changing conditions.

## Synthesis and conceptualization of published articles

The articles nestled within this Research Topic offer a panorama of insights that illuminate the interwoven narrative of coastal ecosystems and aquatic physiology in a changing climate. The Australasian Snapper *Chrysophrys auratus,* a keystone species of commercial and recreational importance, defies expectations by displaying metabolic resilience in the face of marine heatwaves and hypoxia. This resilience emerges as a testament to the intricate balance between thermal plasticity and chronic exposure, uncovering a nuanced physiological response that defies conventional predictions Bowering et al.

Moving from finfish to flora, the seagrass *Posidonia oceanica* emerges as a sentinel of thermal vulnerability. Through the lens of ontogeny-specific thermal sensitivity, the intricate ballet between respiration and net production is unveiled across various life stages. This dance, dictated by temperature and the delicate balance of physiological functions, reflects the adaptability of seagrasses in the face of climatic upheaval Rinaldi et al.

Zooming into the molecular scale, bivalves serve as molecular storytellers. The journey of *Aequiyoldia* bivalves across shifting climates offers a glimpse into the intricate interplay of gene expression, hypoxia, and temperature. The script written in these molecular signatures underscores the role of adaptation and plasticity in shaping the invasibility of new habitats Martínez et al.

Meanwhile, Antarctic marine invertebrates provide a surprising illustration of escape responses as thermal survival mechanisms.

In the thermally stable Southern Ocean, where mobility plays a rather subordinate role in escaping temperature stress, the link between acute tolerance limits and escape response comes into focus. These results point to the complex adaptive ballet that organisms undergo depending on their habitat and their chances of survival in a changing world Morley et al.

## Concluding remarks

This Research Topic of articles extends an invitation to peer into the heart of coastal/benthic realms, where physiology and environment dance a delicate duet. It beckons us to celebrate the tenacity of marine life and contemplate the symphony of resilience that unfolds against the backdrop of climate change. In this narrative, the dynamic equilibrium of fluctuating environments assumes a hallowed role, reminding us that amidst the storm of change, life’s resilience is both the orchestra and the audience. Circadian rhythms, migrations, and behaviours emerge as symphonies of anticipation, allowing species to thrive amidst the fluctuations (Frölicher et al., 2020). Yet, the harmonious resonance of these mechanisms faces discord as human-induced change disrupts the age-old cues, challenging the very foundations of survival.

In the grand tapestry of existence, the interplay of climate and physiology weaves a story of adaptation and survival. As coastal ecosystems continue to evolve under the pressures of a changing climate, it is imperative that we embrace the lessons offered by these fluctuating environments (Fusi et al., 2022). The ocean’s symphony, composed of intricate rhythms and harmonies, reminds us that life’s resilience is not a static trait but an ever-evolving dance of survival. By studying the impact of climate change on coastal environmental variability and aquatic physiology, we unearth the secrets of this dance—a dance that holds the key to understanding the intricate web of life and safeguarding its future in a world of fluctuations.
